# Comparing Idiopathic Chronic Fatigue and Myalgic Encephalomyelitis/Chronic Fatigue Syndrome (ME/CFS) in Males: Response to Two-Day Cardiopulmonary Exercise Testing Protocol

**DOI:** 10.3390/healthcare9060683

**Published:** 2021-06-05

**Authors:** C. (Linda) M. C. van Campen, Frans C. Visser

**Affiliations:** Stichting Cardiozorg, Planetenweg 5, 2132 HN Hoofddorp, The Netherlands; fransvisser@stichtingcardiozorg.nl

**Keywords:** chronic fatigue syndrome, cardiopulmonary exercise testing, VO_2_ peak, ventilatory threshold, VO_2_ AT, RER, myalgic encephalitis, workload, idiopathic chronic fatigue

## Abstract

(1) Introduction: Multiple studies have shown that peak oxygen consumption is reduced in the majority of myalgic encephalomyelitis/chronic fatigue syndrome (ME/CFS )patients, using the gold standard for measuring exercise intolerance: cardiopulmonary exercise testing (CPET). A 2-day CPET protocol has shown different results on day 2 in ME/CFS patients compared to sedentary controls. No comparison is known between ME/CFS and idiopathic chronic fatigue (ICF) for 2-day CPET protocols. We compared ME/CFS patients with patients with chronic fatigue who did not fulfill the ME/CFS criteria in a male population and hypothesized a different pattern of response would be present during the 2nd day CPET. (2) Methods: We compared 25 male patients with ICF who had completed a 2-day CPET protocol to an age-/gender-matched group of 26 male ME/CFS patients. Measures of oxygen consumption (VO_2_), heart rate (HR), systolic and diastolic blood pressure, workload (Work), and respiratory exchange ratio (RER) were collected at maximal (peak) and ventilatory threshold (VT) intensities. (3) Results: Baseline characteristics for both groups were similar for age, body mass index (BMI), body surface area, (BSA), and disease duration. A significant difference was present in the number of patients with fibromyalgia (seven ME/CFS patients vs. zero ICF patients). Heart rate at rest and the RER did not differ significantly between CPET 1 and CPET 2. All other CPET parameters at the ventilatory threshold and maximum exercise differed significantly (*p*-value between 0.002 and <0.0001). ME/CFS patients showed a deterioration of performance on CPET2 as reflected by VO_2_ and workload at peak exercise and ventilatory threshold, whereas ICF patients showed improved performance on CPET2 with no significant change in peak workload. (4) Conclusion: This study confirms that male ME/CFS patients have a reduction in exercise capacity in response to a second-day CPET. These results are similar to published results in male ME/CFS populations. Patients diagnosed with ICF show a different response on day 2, more similar to sedentary and healthy controls.

## 1. Introduction

Myalgic Encephalomyelitis/Chronic Fatigue Syndrome (ME/CFS) is a serious and potentially disabling chronic disease [1,2,3,4]. Little is known about subjects with chronic fatigue who do not fulfill the criteria for ME/CFS. Do they have incipient ME/CFS or is this a different disease process?

One of the defining characteristics of patients with ME/CFS is exercise intolerance combined with a prolonged recovery and exacerbation of symptoms termed post-exertional malaise (PEM) [5,6]. The increase in fatigue and other symptoms following increases in activity is thought to involve metabolic abnormalities of skeletal muscles as well as central nervous system abnormalities [5,7,8,9,10,11,12].

Disability in ME/CFS is multi-dimensional, with social, physical, mental, training, and labor dimensions [4]. A measurement of peak oxygen consumption might indicate the severity of physical activity intolerance [13,14,15,16,17,18,19,20,21,22,23,24]. Cardiopulmonary exercise test (CPET) values of males and females differ amongst other things due to body composition [25,26,27,28]. As the majority of ME/CFS patients are female [4], limited studies with limited numbers on male ME/CFS patients have been reported [17,29,30]. Nelson et al. reported on a combined female/male patient group with a healthy subject control group: five male patients and seven male healthy subjects were included in a 10 patient versus 17 healthy control comparison [29]. Keller studied five males in a combined 22 ME/CFS patient group and used no control group [17]. We recently reported on a relatively large study that included 25 male ME/CFS patients; however, no control group was present [30]. Reports in the literature have shown that, especially on the second-day performance, abnormalities indicative for exercise intolerance abnormalities of ME/CFS become more obvious [17,20,21,29]. In 2-day CPET protocols, no comparisons have been made between ME/CFS patients and those who do not fulfil the criteria ME/CFS and therefore are diagnosed with idiopathic chronic fatigue (ICF) [1,3].

Therefore, the aim of this study was to study the effect of a 2-day CPET protocol in a male ME/CFS patient population to study the effects of 2-day maximal exercise on oxygen consumption at the ventilatory threshold and peak exercise and compare the results with a population diagnosed with ICF.

## 2. Materials and Methods

### 2.1. Participants

From a database of patients evaluated for ME/CFS over the period from June 2010 to October 2019 at the Cardiozorg (a specialist cardiology clinic), we selected male patients who had undergone a 2-day cardiopulmonary exercise test (CPET) protocol for the quantification of exercise intolerance in a clinical situation of excessive fatigue. We identified males who satisfied the criteria for ME/CFS, comparing them with male patients not fulfilling the criteria and who had been diagnosed with idiopathic chronic fatigue (ICF) [1,3]. Patients were included in each group provided that no alternative explanations for the symptoms were found. We limited this study to male patients because of differences in peak oxygen consumption between males and females and possible gender differences in the clinical phenotype of the disease. We have reported results on females separately [31,32,33,34]. No important co-morbidities were present. Male ME/CFS patients graded as having severe ME/CFS according to the international consensus criteria (ICC) were excluded from this analysis, as none of the ICF patients of the control group had a disease of comparable severity.

Of the 111 male patients undergoing CPET over the study period, 35 were diagnosed with idiopathic chronic fatigue of whom 25 underwent a 2-day CPET protocol. We excluded 33 male ME/CFS male patients who had only completed a single-day CPET and 10 others who had more than one test, but not on 2 consecutive days. Seven severe male ME/CFS patients were excluded, leaving 26 male patients with data from a 2-day CPET protocol available for analysis.

All patients gave written informed consent to analyze their data. The use of clinical data for descriptive studies was approved by the ethics committee of the Slotervaart Hospital, the Netherlands.

### 2.2. Cardiopulmonary Exercise Testing (CPET)

The CPET was performed according to the protocol as described elsewhere and used in other studies [30,35,36]. Details are described in Appendix A.

### 2.3. Statistical Analysis

Data were analyzed using the statistical package of Graphpad Prism version 8.4.2 (Graphpad software, La Jolla, CA, USA). All continuous data were tested for normal distribution using the D’Agostino-Pearson omnibus normality test and were presented as mean (SD) or as median with the IQR, where appropriate. Nominal data (fibromyalgia and severity/disability) were compared using the chi-square test. For continuous data, groups were compared using the paired *t*-test/Wilcoxon matched-pairs signed-rank test or unpaired *t*-test/Mann–Whitney test where appropriate. A *p*-value of <0.01 was considered significantly different.

## 3. Results

### 3.1. Baseline Characteristics

Table 1 shows similar age, height, weight, BMI, and disease duration and severity between groups. Only the prevalence of fibromyalgia differed: seven males in the ME/CFS patient group and no males in the ICF patient group (*p* < 0.01).

### 3.2. Results of the 2-Day Cardiopulmonary Exercise Test Protocol

**Figure 1:**
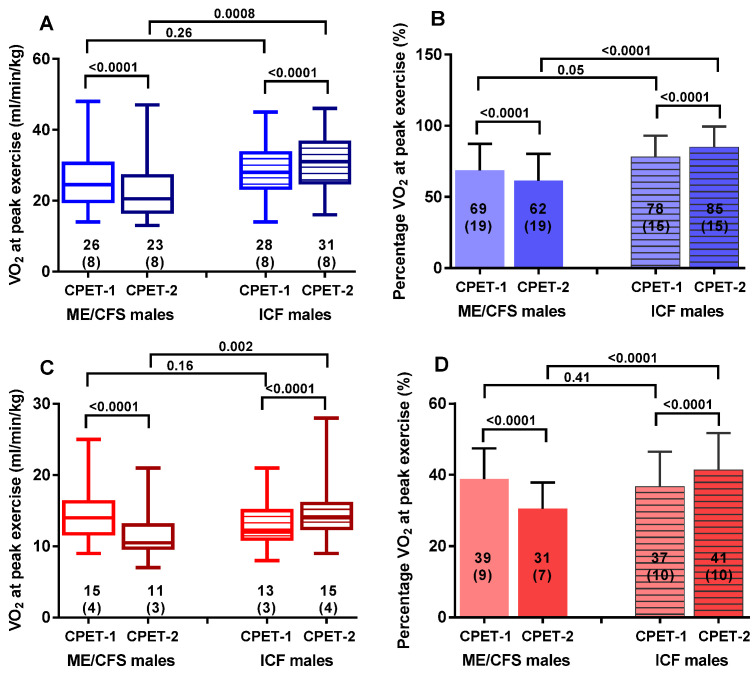
Peak exercise values and values at the ventilatory threshold for CPET-1 and CPET-2. Panel (**A**): peak oxygen consumption, panel (**B**): percent predicted peak oxygen consumption, panel (**C**): oxygen consumption at the ventilatory threshold, and panel (**D**): percent predicted oxygen consumption at the ventilatory threshold. Legend Figure 1: CPET: cardiopulmonary exercise test; VT: anaerobic or ventilatory threshold; VO_2_: oxygen consumption; ME/CFS: myalgic encephalomyelitis/chronic fatigue syndrome; ICF: idiopathic chronic fatigue.

**Figure 2:**
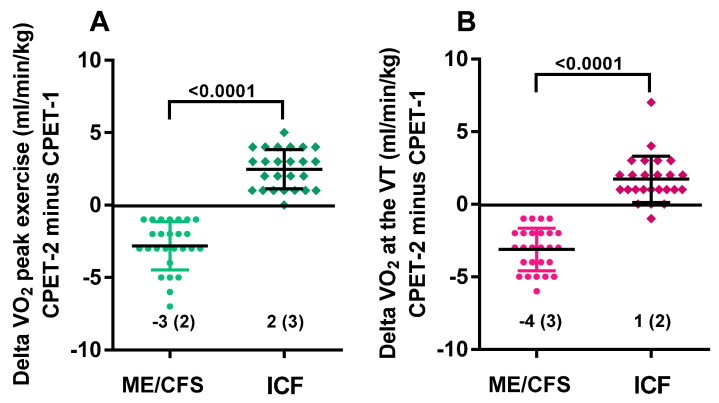
The range of absolute differences for peak VO_2_ (**A**) and VO_2_ at the ventilatory threshold (**B**) for ME/CFS and ICF patients. Legend Figure 2: CPET: cardiopulmonary exercise test; ME/CFS: myalgic encephalomyelitis/chronic fatigue syndrome; ICF: idiopathic chronic fatigue; VO_2_: oxygen consumption; VT: ventilatory threshold.

Table 2 shows the parameters of the CPET on day 1 and day 2 for male ME/CFS patients on the left side of the table and for male ICF patients on the right side of the table. Within each diagnostic group, except for heart rate at rest (*p* = 0.53 and *p* = 0.76, respectively, for ME/CFS and ICF patients) and RER (*p* = 0.08 and *p* = 0.31, respectively, for ME/CFS and ICF patients), all parameters at the ventilatory threshold and at peak exercise differed significantly between day 1 and day 2 (*p* all <0.0001 for ME/CFS patients and *p* ranging between 0.002 and <0.0001 for ICF patients). Figure 1 shows the graphic representation for absolute (panel A) and percent predicted (panel B) peak oxygen consumption and absolute (panel C) and percent predicted (panel D) oxygen consumption at the ventilatory threshold. Within each group, all values differed significantly from day 1 to day 2 (all *p* < 0.0001). While day-1 values did not differ between ME/CFS and ICF patients, day-2 values differed significantly between those two groups (*p* ranging between <0.001 and <0.0001). Figure 2 illustrates the absolute differences between day 1 and day 2 for the peak oxygen consumption and oxygen consumption at the ventilatory threshold between day-1 and day-2. ICF participants improved their CPET performance, while ME/CFS participants had worse results.

**Figure 3:**
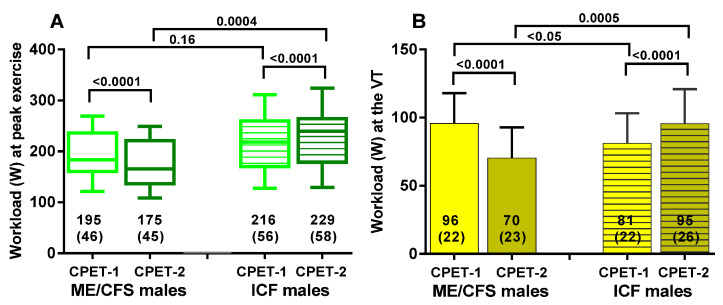
The workload at peak exercise for CPET-1 and CPET-2 panel (**A**) and at the ventilatory threshold for CPET-1 and CPET-2 panel (**B**). On the left side, the values of day 1 and day 2 are shown for male ME/CFS patients (clear boxes/columns); on the right side, the values of day 1 and day 2 for male ICF patients (striped boxes/columns). Workload at peak exercise for CPET-1 and CPET-2 panel (**A**) and at the ventilatory threshold for CPET-1 and CPET-2 panel (**B**). Legend Figure 3: CPET: cardiopulmonary exercise test; ME/CFS: myalgic encephalomyelitis/chronic fatigue syndrome; ICF: idiopathic chronic fatigue; VT: ventilatory threshold.

**Figure 4:**
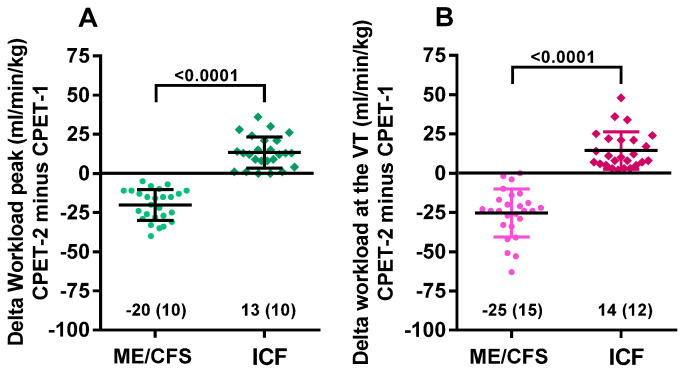
The range of absolute differences for peak workload (**A**) and workload at the ventilatory threshold (**B**) for ME/CFS and ICF patients. Legend Figure 4: CPET: cardiopulmonary exercise test; ME/CFS: myalgic encephalomyelitis/chronic fatigue syndrome; ICF: idiopathic chronic fatigue; VT: ventilatory threshold.

Figure 3 shows the within-group and between-group changes in workload at peak exercise and at the ventilatory threshold for both ME/CFS and ICF patients. Day 1 and day 2 differed significantly in both groups (*p* all <0.0001). A higher workload at the ventilatory threshold in day-1 ME/CFS was found (*p* < 0.05); compared to ICF, this difference was larger with comparison of day-2 results (*p* = 0.0005). Figure 4 shows the difference in both workload measures when day 1 and day 2 were compared in the two groups. Both differed significantly (*p* both <0.0001).

Table 3 shows the between-group comparison of ME/CFS patients with ICF patients at day 1 and day 2, respectively. On day 1, ICF patients had a significantly lower peak oxygen consumption compared to ME/CFS patients (*p* < 0.0001) and also a lower workload at the ventilatory threshold (*p* < 0.05). None of the other parameters differed on day 1 between ME/CFS and ICF patients. On day 2, except for a non-significant difference in heart rate at rest (*p* = 0.07), all parameters differed significantly between ME/CFS and ICF patients (*p* ranging between 0.001 and <0.0001 for parameters at the ventilatory threshold and *p* ranging between 0.002 and <0.0001 for parameters at peak exercise).

## 4. Discussion

The main finding of this study was that with specific differences in peak exercise values as well as values at the ventilatory threshold, where reductions of those values are considered a disease-specific response in ME/CFS patients, the response in ICF patients was more similar to the response of sedentary controls on the second day of a 2-day CPET protocol.

ME/CFS patients who complete a consecutive-day CPET have a reduction in exercise performance when compared to controls [20,21,22,29,37], notably involving a decrease in peak oxygen consumption and oxygen consumption at the ventilatory threshold compared to controls. No large studies of males have been published: two studies combined males and females [17], with only one with a combination of male and female control subjects [29]. Only one “larger” 2-day CPET protocol in males has been published, but with no control group [30]. No 2-day exercise protocols have been described in patients not fulfilling the ME/CFS criteria and therefore having a diagnosis of idiopathic chronic fatigue. The present study is the first—besides the publication on females—to show that patients with the diagnosis of idiopathic chronic fatigue show a different response on a 2-day CPET protocol than ME/CFS patients do, suggesting the abnormalities found in a 2-day exercise protocol in ME/CFS patients are a unique feature of the disease. The findings of the lower VO2 at peak exercise on the second day in ME/CFS patients have been suggested to be the result of metabolic abnormalities, rather than due to deconditioning [21,38]. It may represent an early sign of post-exertional malaise (PEM) [4], one of the essential symptoms of the disease. The general improvement on day 2 for patients with idiopathic chronic fatigue, who are without the symptom of post-exertional malaise and who on the second day of CPET-show a pattern similar to sedentary and healthy controls, suggesting that the changes found on day 2 in ME/CFS patients are disease-specific.

### 4.1. Cardiopulmonary Exercise Testing in Idiopathic Chronic Fatigue: Comparison to Literature

One report on a large sample of patients (females and males with idiopathic chronic fatigue) is present in current literature, but the report was on a one-day CPET test, with comparisons with ME/CFS patients and healthy controls of both genders [23]. This study reported a single-day CPET protocol comparison with seven male healthy controls, 25 male ME/CFS patients, and 51 male ICF patients. We report the results of males in this to allow comparisons of results with our present study results. At-rest ICF males had significantly lower weight than the other two groups: 80.0 (12.8) kg in ICF males compared to 88.9 (15.7) kg in ME/CFS males and 94.3 (13.9) kg in healthy males (ANOVA *p* = 0.005), as well as BSA: 2.02 (0.17) m^2^ in ICF males compared to 2.12 (0.21) m^2^ in ME/CFS males and 2.19 (0.16) m^2^ in healthy males (ANOVA *p* = 0.016). At rest, no differences were found in heart rate, oxygen consumption, or RER. At the ventilatory threshold, an ordinary one-way ANOVA was not significant for VO2: 11.8 (2.8) mL/min/kg for male ME/CFS patients, 13.4 (3.3) mL/min/kg for male ICF patients, and 13.7 (3.1) for healthy male subjects (*p* = 0.093), but no difference was found for the heart rate at the ventilatory threshold or the RER at the ventilatory threshold. At peak exercise, an ordinary one-way ANOVA was significant for VO_2_: 24.0 (7.2) mL/min/kg for male ME/CFS patients, 28.9 (7.1) mL/min/kg for male ICF patients, and 27.3 (3.7) for healthy male subjects (*p* = 0.019). This was similar for percent predicted VO_2_: 73.9 (17.5)%, 83.4 (19.2)%, and 96.2 (11.4)%, respectively (*p* = 0.011). Peak heart rate was not significantly different, as well as RER at peak exercise. No post-hoc statistical information was present whether ME/CFS and ICF results were significantly different between those groups. In the present study, VO_2_ at the ventilatory threshold for day 1 was 13 mL/min/kg for ME/CFS females and 11 mL/min/kg for ICF females, similar to the results of the study of Vermeulen et al. The peak VO_2_ were between 21 and 22 mL/min/kg for ME/CFS and ICF females, which was in the same range.

### 4.2. Limitations

First, no male sedentary controls were included for comparison in this study. Information on sedentary/healthy controls has been described by other research groups. As a comparison group, we used males not fulfilling ME/CFS criteria, namely those diagnosed with idiopathic chronic fatigue (ICF). Second, this was not a prospective trial, as most patients underwent consecutive day CPET for clinical management reasons. Third, differences between the previously discussed studies and the present study might be in the demographic characteristics and illness severity of the study population, but also in the exact methodology of the CPET used in the different study centers. Finally, reference values for predicted VO_2_ can differ between studies as well.

## 5. Conclusions

This study in male ME/CFS patients compared with male ICF patients shows that exercise capacity expressed in peak VO_2_, VO_2_ at the ventilatory threshold, and workload both at peak and at the ventilatory threshold show a different pattern from day 1 to day 2 for the two patient groups. The ICF group might respond to exercise training, whereas ME/CFS might not.

## Figures and Tables

**Table 1:** Baseline criteria.

	Male ME/CFS (*n* = 26)	Male ICF (*n* = 25)	*p*-Value
Age (years)	44 (12)	43 (10)	0.63
Height (cm)	183 (7)	184 (8)	0.73
Weight (kg)	85 (15)	81 (11)	0.31
BMI (kg/m^2^)	25.5 (4.0)	224.2 (2.9)	0.18
BSA (m^2^)	1.7 (0.2)	1.6 (0.2)	0.40
Disease duration (years)	12 (8)	12 (9)	0.97
Disease severity grade ½ *	10/16 (38/62%)	16/9 (64/36%)	0.07 *
Fibromyalgia present *	7	0	0.005

Disease severity grading: 1 is mild disease and 2 is moderate disease (1); BMI: body mass index (DuBois formula); BSA: body surface area. Mean (SD), analysis with unpaired *t*-test; Median (IQR); * analysis with Chi-square.

**Table 2:** CPET-1 and CPET-2 variables for male ME/CFS patients (left side) and male ICF patients (right side).

	ME/CFS Males (*n* = 26)			ICF Males (*n* = 25)		
**Peak Exercise**	**CPET-1**	**CPET-2**	***p*-Value**	**CPET-1**	**CPET-2**	***p*-Value**
VO_2_ peak (mL/min/kg)	26 (8)	23 (8)	<0.0001	28 (8)	31 (8)	<0.0001
%pred VO_2_ peak	69 (19)	62 (19)	<0.0001	78 (15)	85 (15)	<0.0001
HR rest (bpm)	79 (12)	78 (10)	0.53	83 (11)	83 (13)	0.76
HR peak (bpm)	148 (23)	138 (24)	<0.0001	160 (22)	165 (20)	0.002
Workload peak (Watts)	195 (46)	175 (45)	<0.0001	216 (56)	229 (58)	<0.0001
RER peak	1.1 (0.1)	1.1 (0.1)	0.08	1.2 (0.1)	1.2 (0.1)	0.31
**Ventilatory Threshold**	**CPET-1**	**CPET-2**	***p*-Value**	**CPET-1**	**CPET-2**	***p*-Value**
VO_2_ VT (mL/min/kg)	15 (4)	11 (3)	<0.0001	13 (3)	15 (4)	<0.0001
%pred VO_2_ VT	39 (9)	31 (7)	<0.0001	37 (10)	41 (10)	<0.0001
HR VT (bpm)	109 (15)	96 (12)	<0.0001	103 (12)	109 (14)	0.0002
Workload VT (Watts)	96 (22)	70 (23)	<0.0001	81 (22)	95 (26)	<0.0001

VT: ventilatory threshold; CPET: cardiopulmonary exercise test; HR: heart rate; pred: predicted; RER: respiratory exchange ratio; VO_2_: oxygen consumption.

**Table 3:** CPET-1 variables in ME/CFS compared to ICF males (left side) and CPET-2 variables in ME/CFS compared to ICF males (right side).

	Cpet Day-1			Cpet Day-2		
**Peak Exercise**	**ME/CFS**	**ICF**	***p*-Value**	**ME/CFS**	**ICF**	***p*-Value**
VO_2_ peak (mL/min/kg)	26 (8)	28 (8)	<0.0001	23 (8)	31 (8)	0.0008
%pred VO_2_ peak	69 (19)	78 (15)	0.05	62 (19)	85 (15)	<0.0001
HR rest (bpm)	79 (12)	83 (11)	0.21	78 (10)	83 (13)	0.07
HR peak (bpm)	148 (23)	160 (22)	0.07	138 (24)	165 (20)	<0.0001
Workload peak (Watts)	195 (46)	216 (56)	0.16	175 (45)	229 (58)	0.0004
RER peak	1.1 (0.1)	1.2 (0.1)	0.08	1.1 (0.1)	1.2 (0.1)	0.002
**Ventilatory Threshold**	**ME/CFS**	**ICF**	***p*-Value**	**CPET-1**	**CPET-2**	***p*-Value**
VO_2_ VT (mL/min/kg)	15 (4)	13 (3)	0.16	11 (3)	15 (4)	0.002
%pred VO_2_ VT	39 (9)	37 (10)	0.41	31 (7)	41 (10)	<0.0001
HR VT (bpm)	109 (15)	103 (12)	0.13	96 (15)	109 (14)	0.001
Workload VT (Watts)	96 (22)	81 (22)	<0.05	70 (23)	95 (26)	0.0005

VT: ventilatory threshold; CPET: cardiopulmonary exercise test; HR: heart rate; pred: predicted; RER: respiratory exchange ratio; VO_2_: oxygen consumption.

**Figure A1:**
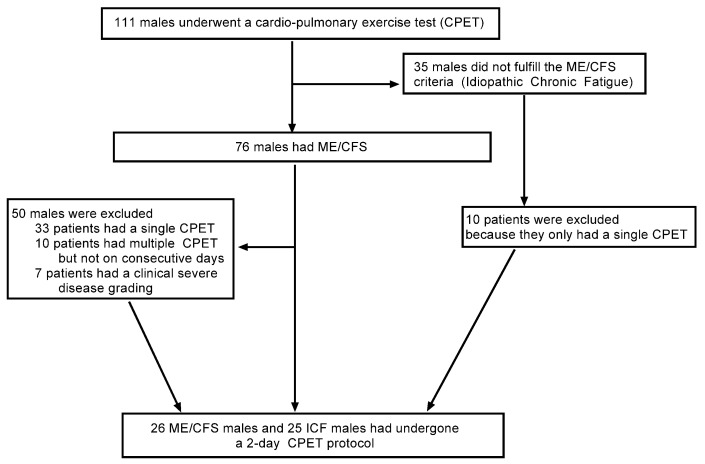
Patient flow. Legend figure: CPET: cardiopulmonary exercise test; ME/CFS: myalgic encephalomyelitis/chronic fatigue syndrome; ICF: idiopathic chronic fatigue.

## Data Availability

The raw data supporting the conclusions of this manuscript will be made available by the authors, without undue reservation, to any qualified researcher.

## Appendix A

### Methodology of the Cardiopulmonary Exercise Test

Patients underwent a symptom-limited exercise test on a cycle ergometer (Excalibur, Lode, Groningen, The Netherlands) according to a previously described protocol [30,35,36]. A RAMP workload protocol was used varying between 10–30 Watt/min increases, depending on sex, age, and expected exercise intolerance. Oxygen consumption (VO_2_), carbon dioxide release (VCO_2_), and oxygen saturation were continuously measured (Cortex, Procare, The Netherlands), and displayed on screen using Metasoft software (Cortex, Biophysic Gmbh, Germany). An ECG was continuously recorded, and blood pressures were measured continuously using the Nexfin device (BMEYE, Amsterdam, The Netherlands) [39]. Cycle seat height was positioned to approximately 175° of knee extension, and the same seat height was used for both tests. Expired gases were collected breath-by-breath through a two-way breathing valve and analyzed using open circuit spirometry. The metabolic measurement system (Cortex, Biophysic Gmbh, Germany) was calibrated before each test with ambient air, standard gases of known concentrations, and a 3-L calibration syringe. Ventilatory threshold (VT) is an analog of anaerobic threshold and was identified from expired gases using the V-Slope algorithm in the metabolic measurement system software. Ventilatory or anaerobic threshold is the exercise intensity at which metabolism transitions toward increased anaerobic energy production. The same trained investigator performed visual assessment and confirmation of the algorithm-derived VT. Testing took place in a controlled environment with temperature range of 20–24 °C and 15–60% relative humidity. The test was supervised by an experienced cardiologist. Patients were encouraged by standard phrases each minute to perform maximally to the point of exhaustion. The mean of the VO_2_ measurements of the last 15 s before ending the exercise (peak VO_2_) was taken and expressed as a percentage of the normal values of a population study: %peak VO_2_ [40]. We assessed the mean respiratory exchange ratio (RER; VCO_2_/VO_2_) of the last 15 s to determine the influence of this measure of maximal effort on the results. Immediately after the test, the attending cardiologist noted the primary reason for termination of the exercise and judged whether motivation and efforts during exercise were optimal for the individual patient.
